# Facelift thyroid surgery: a systematic review of indications, surgical and functional outcomes

**DOI:** 10.1186/s40463-023-00624-x

**Published:** 2023-04-10

**Authors:** Jérôme R. Lechien, Piero M. Fisichella, Giovanni Dapri, Jonathon O. Russell, Stéphane Hans

**Affiliations:** 1 Robotic Surgery Study Group of Young-Otolaryngologists of the International Federations of Oto-Rhino-Laryngological Societies (YO-IFOS), Paris, France; 2 Department of Otolaryngology - Head & Neck Surgery, Foch Hospital, School of Medicine, UFR Simone Veil, Université Versailles Saint-Quentin-en-Yvelines (Paris Saclay University), Paris, France; 3 Department of Human Anatomy and Experimental Oncology, Faculty of Medicine, UMONS Research Institute for Health Sciences and Technology, University of Mons (UMons), Mons, Belgium; 4 Department of Otolaryngology, Elsan Hospital, Paris, France; 5 AbbVie Clinical Pharmacology Research Unit, Chicago, IL USA; 6 Department of Minimally Invasive General and Oncologic Surgery, Humanitas Gavazzeni University Hospital, Bergamo, Italy; 7 International School Reduced Scar Laparoscopy, Bergamo, Italy; 8 Division of Head and Neck Endocrine Surgery, Department of Otolaryngology-Head and Neck Surgery, Johns Hopkins University, School of Medicine, Baltimore, MD USA; 9 Department of Otorhinolaryngology and Head and Neck Surgery, CHU Saint-Pierre, Brussels, Belgium

**Keywords:** Thyroid, Facelift, Hairline, Robotic, Endoscopic, Minimal, Invasive, Surgery, Otolaryngology, Head neck

## Abstract

**Objective:**

To investigate indications, surgical and functional outcomes of robotic or endoscopic facelift thyroid surgery (FTS) and whether FTS reported comparable outcomes of other surgical approaches.

**Data sources:**

PubMed, Cochrane Library, and Scopus.

**Review methods:**

A literature search was conducted about indications, clinical and surgical outcomes of patients who underwent FTS using PICOTS and PRISMA Statements. Outcomes reviewed included age; gender; indications; pathology; functional evaluations; surgical outcomes and complications.

**Results:**

Fifteen papers met our inclusion criteria, accounting for 394 patients. Endoscopic or robotic FTS was carried out for benign and malignant thyroid lesions, with or without central neck dissection. Nodule size and thyroid lobe volume did not exceed 6, 10 cm, respectively. FTS reported comparable outcome with transaxillary or oral approaches about operative time, complication rates or drainage features. The mean operative time ranged from 88 to 220 min, depending on the type of surgery (endoscopic vs robotic hemi- or total thyroidectomy). Conversion to open surgery was rare, occurring in 0–6.3% of cases. The most common complications were earlobe hypoesthesia, hematoma, seroma, transient hypocalcemia and transient recurrent nerve palsy. There was an important disparity between studies about the inclusion/exclusion criteria, surgical and functional outcomes.

**Conclusion:**

FTS is a safe and effective approach for thyroid benign and malignant lesions. FTS reports similar complications to conventional thyroidectomy and excellent cosmetic satisfaction.

**Graphical abstract:**

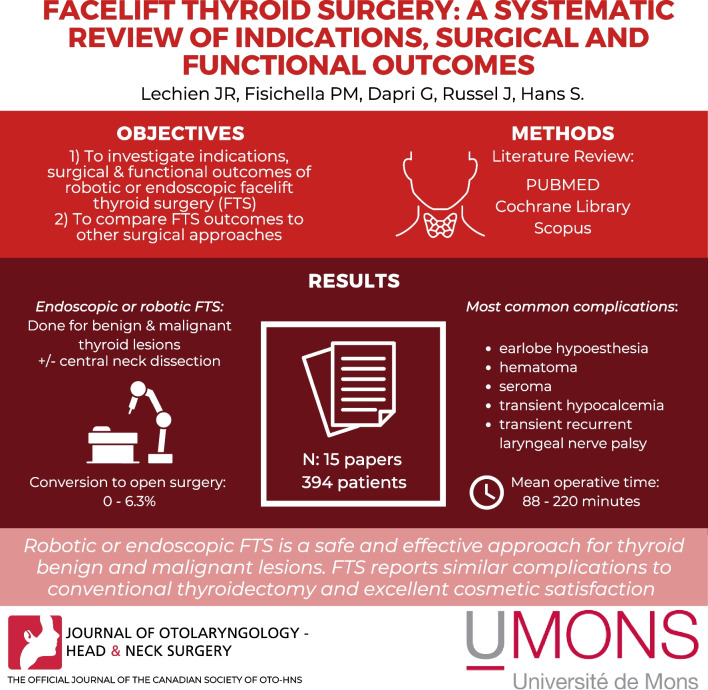

**Supplementary Information:**

The online version contains supplementary material available at 10.1186/s40463-023-00624-x.

## Introduction

Robotic or endoscopic surgery gained in popularity for the treatment of small benign or malignant lesions of the thyroid gland over the past two decades [1, 2]. Several surgical approaches were developed, including axillary, transoral or retroauricular (facelift or necklift) surgeries [2, 3]. The most important argument to develop such approaches remains the desire to avoid or hide visible neck scarring, while ensuring similar or better post-operative morbidity outcomes. Retroauricular facelift approach, also called cephalic access thyroid surgery, is an endoscopic or robotic approach through the hairline, allowing the realization of hemi- or total thyroidectomy [2]. This approach was described as safe, feasible and was associated with few post-operative complications according to many studies [4–6]. However, indications, clinical and surgical outcomes may substantially vary from one study to another, leading to conflicting ideas.

In this systematic review, we aimed to investigate indications, surgical and functional outcomes of robotic or endoscopic facelift thyroid surgery (FTS) and whether FTS approaches reported comparable outcomes of other surgical approaches.

## Material and methods

The criteria for consideration of study inclusion were based on the population, intervention, comparison, outcome, timing and setting (PICOTS) framework [7]. Three authors (JRL, GD & SH) independently reviewed and extracted data of studies regarding a modified PRISMA checklist for systematic reviews [8].

### Patient population

Prospective and retrospective, controlled or uncontrolled, studies including patients who underwent robotic or endoscopic facelift thyroid surgery (FTS) were considered. Studies were published in English, Spanish or French peer-reviewed journals between January 2000 and July 2022. Only studies reporting data for more than 10 individuals were considered. Inclusion or exclusion criteria and surgical approaches had to be specified in studies. Authors (JRL, GD & SH) classified the study regarding the levels of evidence (I–V) [9].

### Intervention and comparison

The following surgical approaches were considered in comparative studies: conventional thyroidectomy (CT), transaxillary thyroid surgery (TAT) or transoral thyroid surgery (TO, Additional file 1: Appendix 1).

### Outcomes

Authors investigated the following outcomes: study design; country; period of patient inclusion; type of surgery (i.e. hemi- versus total thyroidectomy, neck dissection, robotic/endoscopic FTS, CT, TAT, TO); number of patients; body mass index (BMI); gender ratio; mean or median age; pathological outcomes (benign *versus* malignant lesions, stages, tumor/nodule size); surgical outcomes (e.g. operative step times, drain, amount of drainage, blood loss, conversion rate); complications, cosmetic and functional outcomes. Two authors (JRL & SH) used the MINORS score for the bias analysis [10]. MINORS is a methodological index for non-randomised studies in which the items were scored 0 if not reported; 1 when reported but inadequate; and 2 when reported and adequate. The global ideal MINORS score is 16 for non-comparative studies and 24 for comparative studies.

### Timing and setting

There was no criteria for specific stage or timing in the ‘disease process’ of the included populations.

### Search strategy

The publication search was conducted with PubMED, Scopus, and Cochrane databases. The databases were screened for abstracts and titles referring to the description of outcomes of patients benefiting from robotic or endoscopic FTS. The three authors analyzed full texts of selected studies. Results of the search strategy were reviewed for relevance and the reference lists of these publications were examined for additional pertinent studies. There were no discrepancies in synthesized data among the three authors. The following keywords were considered and combined: ‘thyroid’; ‘thyroidectomy’; ‘facelift’; ‘cephalic’; ‘cancer’; ‘nodule’; ‘robotic’; ‘endoscopic’; ‘approach’ and ‘outcomes’.

## Results

**Fig. 1:**
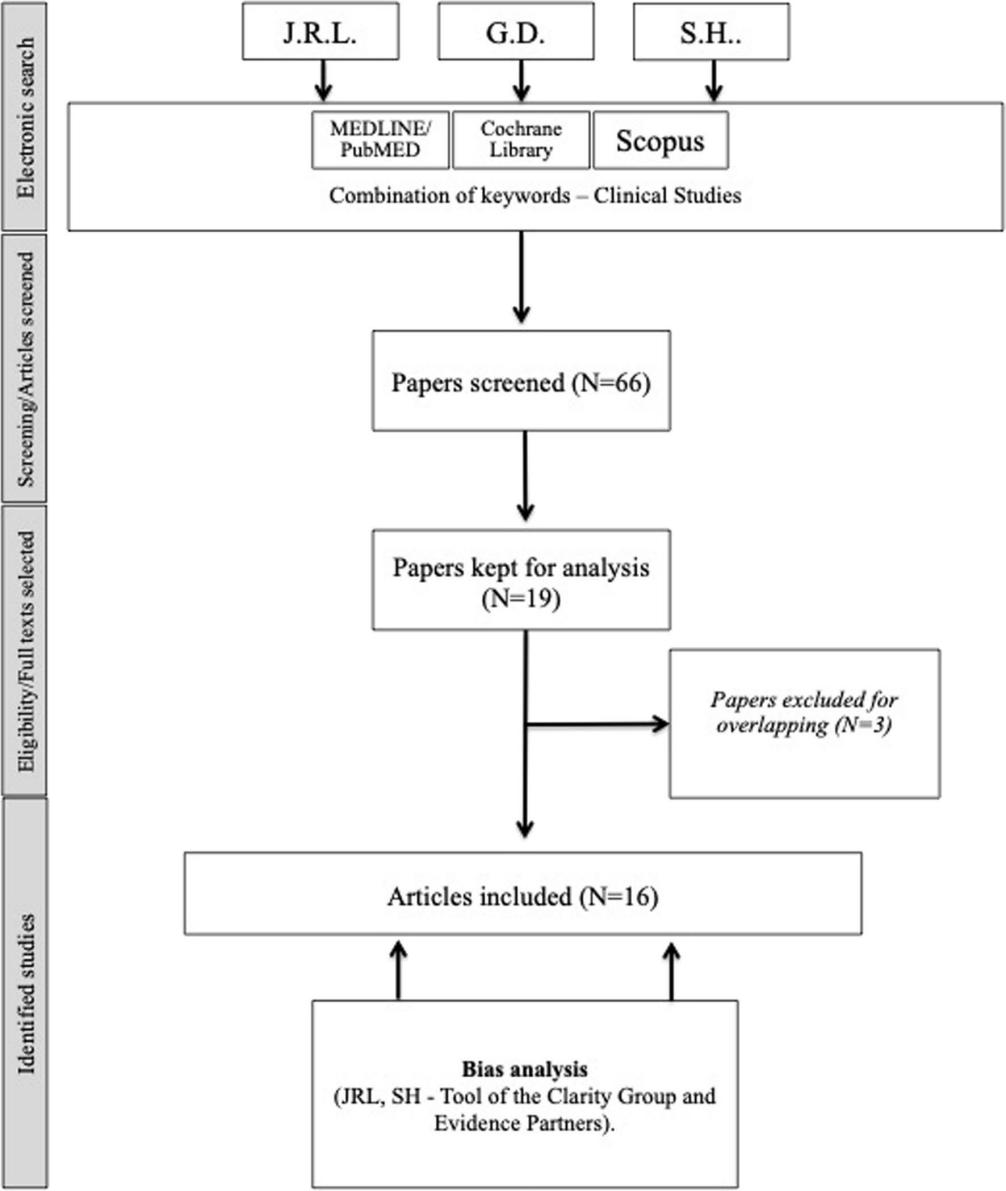
Modified PRISMA flowchart

From the 66 publications identified, 16 papers met our inclusion criteria (PRISMA flowchart, Fig. 1) [11–26]. There were 5 controlled prospective studies (EBL: IIb), 6 uncontrolled prospective studies (EBL: IIIb) and 5 retrospective chart-reviews (EBL: IV; Additional file 1: Appendix 2). The following world regions were represented in studies: Korea (N = 7), United States (N = 7), Germany (N = 1) and India (N = 1). Three papers were excluded because patient overlapping [4, 5, 27]. The present study includes 431 patients. According to studies that reported gender data, the female/male ratio was 319/68. The mean age of patients ranged from 23 to 57 years (Additional file 1: Appendix 2). The mean BMI ranged from 23.6 to 27.4 in patients benefiting from endoscopic or robotic FTS. Among comparative studies, the following thyroid surgery approaches were considered in comparison with FTS: transcervical ‘open’ surgery (N = 5) [18, 19, 22, 23, 25], TAT (N = 6) [12, 16, 21–23, 25], and TO (N = 3) [20, 22, 24]. Endoscopic and robotic FTS were performed in 6 [14, 19, 23–26], and 14 studies [11–13, 15–24, 26], respectively (Additional file 1: Appendix 2).

Note that disparity among included articles in inclusion/exclusion criteria, indications, and outcomes measures precluded statistically pooling the data into a formal meta-analysis, thereby limiting the analysis to a qualitative rather than quantitative summary of the available information.

### Indications, inclusion and exclusion criteria

Five authors [13, 15, 18, 20, 21] only included patients benefiting from hemi-thyroidectomy, while both hemi- and total thyroidectomies were considered in the rest of the studies (Table 1). Most authors considered both suspected benign and malignant lesions, which were categorized by pre-operative fine needle aspiration cytology (FNAC) and ultrasonography findings. Terris et al., only recruited patients with benign or indeterminate (Bethesda class III/IV) cytology [11, 12], while other authors did not include pathological findings in the inclusion criteria [17–19]. The size of the nodule was considered as an important inclusion factor in 7 studies. Two teams included patients with nodule < 4 cm size [13, 17], while Park et al. considered nodule < 2 cm size [14]. The maximal nodule size of the study of Dabas et al. was 3.5 cm [21]. Sung et al. specified that patients with nodule > 5 cm were not included [16]. Authors performed FTS approach in patients exhibiting nodule < 6 cm in three studies [20, 22, 24]. Russell et al. accepted patients with thyroid lobe < 7 cm [20] and < 10 cm, respectively [22]. In the study of Ji et al*.* [24], there were several lesion size criteria, determining inclusion criteria for nodule size (< 6 cm), differentiated carcinoma size (< 4 cm) or benign tumor (< 4 cm) [26]. As reported in Table 1, a myriad of exclusion criteria were considered by authors, the most frequent being history of neck surgery or radiation [11–21, 23, 26], distal metastasis [13, 20, 21, 23], substernal thyroid extension [11, 12, 17, 20, 26], and Graves’ disease or thyroiditis [17, 20, 22]. There was substantial differences between studies about the inclusion [14–16, 24] or exclusion [11, 12, 17, 20–22, 26] of extrathyroidal invasion of lesion (Table 1). The patient BMI was a criterion of exclusion (overweight/obesity) in three studies [11, 12, 14]. Note that authors included both primary and recurrent lesion in two studies [16, 24]. Technical aspects of surgeries were previously reported [28–32] and were not developed in the present paper.

**Table 1:** Inclusion and exclusion criteria of studies

Authors	Country	Inclusion period	Indications	Exclusion
Terris [11] Terris [12]	USA (Augusta)	July 2010–October 2010/February 2011	1. Hemi or total thyroidectomy 2. Growing nodules 3. Small multinodular goiters 4. Follicular neoplasms or unclear malignant potential	1. BMI > 40 2. Previous neck surgery 3. Medical comorbidities contra-indicating anesthesia 4. Abnormal laryngeal function on preoperative laryngoscopy 5. Inability to understand surgical options 6. Acceptance of possible need for conversion 7. Substernal extension, lymphadenopathy or extrathyroidal extension of malignancy
Kandil [13]	USA (New Orleans)	December 2012–March 2013	1. Hemithyroidectomy 2. Enlarging benign, suspicious, or malignant thyroid nodule on thyroid fine needle aspiration biopsy	1. Nodule with > 4 cm size (ultrasonography) 2. Previous neck surgery or radiation 3. Graves' disease with substernal extension 4. Substernal or retropharyngeal goiter 5. Unilateral or bilateral vocal fold immobility at the preoperative laryngoscopy 6. Advanced thyroid malignancy, cervical, or distant nodal metastases
Park [14]	Korea (Seoul)	January 2012–December 2013	1. Hemithyroidectomy for benign nodules or papillary microcarcinoma without extrathyroidal extension	1. Nodule with > 2 cm size (ultrasonography) 2. Previous neck tumor surgery or treatment 3. Obese patient (BMI: NP)
Byeon [15]	Korea (Seoul)	January 2013–Mai 2014	1. Hemi or total thyroidectomy 2. Benign nodules or papillary microcarcinoma without extrathyroidal extension 3. Thyroid carcinomas with neck metastasis	1. No previous history of treatment for thyroid carcinoma 2. Previous neck surgery 3. Recurrent thyroid tumor 4. Cancer with gross invasion to local structures or extensive extrathyroidal capsular extension
Sung [16]	Korea (Seoul)	September 2013–December 2014	1. Hemi or total thyroidectomy 2. Thyroid carcinoma < 4 cm with minimal extrathyroidal extension and small lymph node metastases in the central comportment	1. Nodule with > 5 cm size (ultrasonography) 2. Previous neck surgery or radiation 3. Concurrent lateral neck dissection 4. Cancer with gross invasion to local structures or extensive extrathyroidal capsular extension 5. Completion thyroidectomy
Duke [17]	USA (Augusta)	July 2010–April 2014	1. Hemi or total thyroidectomy 2. Extent of disease appropriate for unilateral surgery	1. Nodule with > 4 cm size (ultrasonography) 2. Previous neck surgery 3. Medical comorbidities contra-indicating anesthesia 4. No obesity 5. Thyroiditis, substernal extension, pathologic lymph node and extrathyroidal extension
Alshehri [26]	USA (New Orleans)	December 2012–Mai 2015	1. Hemi or total thyroidectomy 2. Enlarging nodule < 4 cm 3. American Society of Anesthesia Score 1–2	1. Previous neck surgery/radiation 2. Vocal fold immobility prior surgery 3. Substernal or extrathyroidal extension 4. BMI < 40 5. Advanced thyroid cancer
Song [18]	Korea (Seoul)	January 2015–December 2016	1. Hemithyroidectomy 2. Surgery with or without central neck dissection	1. Gross extrathyroidal extension 2. Previous neck surgery or radiation 3. Concurrent lateral neck dissection 4. Completion hemi or total thyroidectomy 5. Age < 18 or > 70 years 6. Preoperative dysphonia or vocal fold disorders
Ban [19]	Korea (Seoul)	Mai 2014–September 2016	1. Hemi or total thyroidectomy 2. Primary surgery and reoperation	1. Preoperative dysphonia or vocal fold disorders 2. Loss of neuromonitoring during other parts of the surgical procedure aside from thyroid surgery
Russell [20]	USA (New Orleans) (Baltimore)	August 2011–August 2016	1. Hemithyroidectomy 2. Benign or malignant nodules	1. Nodule with > 6 cm size (ultrasonography) 2. Thyroid lobe > 7 cm 3. Previous neck surgery or radiation 4. Cancer with extrathyroidal extension or lymph node metastasis 5. Graves' disease or thyroiditis 6. Substernal extension
Dabas [21]	India (Gurugram)	April 2015–Mai 2016	1. Hemithyroidectomy 2. Solitary thyroid nodule limited to single lobe	1. Nodule with > 3.5 cm size (ultrasonography) 2. Extrathyroidal invasion 3. Previous neck surgery or radiation 4. Bilateral cervical nodes/distant metastasis 5. Vocal fold palsy (before surgery)
Russell [22]	USA (New Orleans) (Baltimore)	December 2012–June 2018 (pathology)	1. Hemi or total thyroidectomy 2. Undetermined thyroid nodules	1. Nodule with > 6 cm size (ultrasonography) 2. Thyroid lobe > 10 cm 3. Previous neck surgery or radiation 4. Cancer with extrathyroidal extension or lymph node metastasis 5. Graves' disease or thyroiditis 6. Substernal extension
Lee [23]	Korea (Seoul)	September 2013–November 2016	1. Hemi or total thyroidectomy 2. Benign or malignant nodules	1. Previous neck surgery or radiation 2. Patients who underwent lateral neck dissection with thyroidectomy 3. Patients with distant metastasis
Ji [24]	Korea (Seoul)	April 2016–September 2018	1. Hemi or total thyroidectomy 2. Benign or malignant nodules	1. Nodule with > 6 cm size (ultrasonography) 2. Differentiated carcinoma > 4 cm 3. Malignant tumor > 2 cm 4. Benign tumor > 4 cm 5. cT4 cancer, maximal extrathyroidal extension 6. Recurrent cancer 7. Preoperative vocal fold palsy
Wirth [25]	Germany (Munich) (Hausham) (Bad Aibling)	Not clearly specified	1. Hemi or total thyroidectomy 2. Benign or malignant nodules	NP

*BMI* body mass index, *N* number, *NP* not provided

### Surgical outcomes

The surgical and functional outcomes are summarized in Table 2. The mean operative time of FTS ranged from 88 to 201 min for hemi-thyroidectomy [11–14, 16, 18, 20, 22] and 132–220 min for total thyroidectomy [15–17, 22, 23, 25, 26] but in both cases, may depend on the approach (robotic *versus* endoscopic) and the realization of lymph node dissection [15, 18] or neck lift surgery (Additional file 1: Appendix 2) [13, 26]. The time of the different surgical steps was assessed in five studies [11, 13, 17, 21, 26]. The pocket dissection time ranged from 40 to 74 min [11, 17, 21], while the docking time ranged from 11 to 17 min [11, 13, 17]. The console time ranged from 15 to 52 min [11, 13, 17, 21, 26]. All of them depending on the type of surgery (hemi *versus* total thyroidectomy) or approach (robotic *versus* endoscopic) but few data are available to provide specific ranges. Conversion rate was low [11, 12, 17, 19–21], with only a single patient reported in the study of Dabas et al. [21].

**Table 2:** Surgical and functional outcomes

Types of outcomes	Subtype of outcomes	N	References
Surgical	Operative total time	12	[11–18, 20, 22, 23, 25, 26]
	Hospital stay	10	[11–15, 17, 18, 20, 21, 25, 26]
	Conversion rate	6	[11, 12, 17, 19–21, 26]
	Drain placement	4	[11, 12, 17, 20]
	Drainage amount	4	[15, 16, 18, 23]
	Pocket dissection time	3	[11, 17, 21]
	Console time	3	[11, 13, 17, 26]
	Blood loss	3	[13, 15, 21, 26]
	Docking time	2	[11, 13, 17]
	Drain duration	2	[14, 15]
	Central neck dissection	1	[18]
	Incision length	1	[17]
Functional	Scar satisfaction	6	[13, 15, 16, 21, 23, 25]
	Subjective voice changes (physician)	2	[14, 21]
	Acoustic parameters	2	[14, 18]
	Postoperative pain	2	[16, 21]
	Neuromonitoring loss/success of use	2	[19, 24]
	Self-perceveid voice change (patient)	1	[18]
	Aerodynamic measurements	1	[18]
	Hypertrophic scaring	1	[20]

Blood loss data during the surgery were reported in three studies, ranging from 22.4 to 45 mL [13, 15, 21]. A surgical drain was thought to be necessary in 7.1, [11] 6.7 [12], 25.6 [17], and 70% [20] of cases and the duration of drainage ranged from one to three days [14, 15]. Drain amount findings were reported in 4 studies. Drain amounts ranged from 122 to 213 mL and depended on both type and approach of surgery [15, 16, 18, 23].

Few studies compared surgical outcomes between several approaches. According to Sung et al. [16], there were no significant operative time differences between robotic FTS and TAT for both hemi- and total thyroidectomy. Authors observed similar findings for average amount of drainage [16]. The operative time was longer for FTS than CT in the study of Song et al. [18], while they did not find differences for amount of drainage between both approaches. Russell et al. [20, 22] compared robotic FTS with TO hemithyroidectomy. Overall, they did not find significant differences between both approaches in operative time [20], but they observed that CT was associated with lower operative time than remote access approaches (FTS, TAT and TO) [22]. The shorter time of CT compared with FTS and TAT was corroborated in the study of Lee et al. [23] where authors reported additionally that TAT and FTS were associated with higher amount of drainage than CT (Additional file 1: Appendix 2). Recently, Wirth et al. showed that the operative time of TAT was significantly longer than those of FTS and CT [25].

The neuromonitoring effectiveness was evaluated in two studies [19, 24]. According to Ji et al. [24], neuromonitoring was effective in 82.6% of cases, and the prevalence of loss of neuromonitoring during the surgery was not different between FTS and CT group [19].

In sum, operative time and surgical drainage may be lower in CT when compared to remote access approaches.

### Complications and comparison between approaches

Complications were described in 15 studies [11–20, 22–26]. The range of incidence of postoperative complications is reported in Table 3. The most prevalent complications consisted of earlobe hypoesthesia, hematoma, seroma, transient hypocalcemia and transient recurrent nerve palsy. About hematoma, only two authors reported distinct prevalence of minor (2.5 & 28.8%) and major (2.5 & 3.4%) hemorrhage; the second requiring surgical revision [25, 26]. Moreover, they observed that minor hemorrhage occurred more frequently in TAT approach compared with FTS and CT, corroborating the observations of Russell et al. [22] Postoperative pain was assessed in two studies and authors reported significant decrease of pain scores in the postoperative few weeks [16, 21]. There were no statistical differences in prevalence of any complication between surgical approaches in the rest of studies.

**Table 3:** Complications reported by authors

Complications	N	References	Range
Transient RNL	13	[11–20, 22, 23, 25, 26]	0–7.1%
Hemorrhage/hematoma	10	[12, 15–20, 22, 23, 25, 26]	0–44.1%
Seroma	9	[11, 13, 15–18, 20, 22, 23, 26]	0–24.1%
Permanent RNL	6	[13–16, 20, 25, 26]	0–3.4%
Earlobe hypoesthesia	5	[11–14, 25]	6.9–100%
Unspecified hypoparathyroidism	4	[11, 17, 18, 23, 26]	0–2.4%
Transient hypoparathyroidism	3	[13, 15, 16]	0–25%
Cellulitis or infections	2	[17, 25, 26]	0–1.1%
Permanent hypoparathyroidism	2	[15, 16]	0%
Spinal nerve lesion	2	[15, 17]	0–1.1%
Mouth corner deviation	1	[15]	5.7%
Chyle leakage	1	[15]	1.1%
Skin flap ischemia	1	[15]	2.3%
Tracheal injury	1	[16]	0%
Postoperative pain	1	[16]	NP*

*N* number of studies where de complication was reported

*Prevalence not provided, only VAS outcomes

### Cosmetic outcomes

The main cosmetic outcomes investigated were cosmetic scar satisfaction [13, 15, 16, 21, 23]. Patients were satisfied of the scar in 100% of cases [13, 15, 16, 21, 23]. Lee et al. [23] reported better cosmetic scar satisfaction scores in patients benefiting from TAT or FTS compared with those who had CT, while Wirth et al. [25] did not find differences between TAT and FTS groups.

### Functional outcomes and comparison between approaches

The main functional outcomes investigated were subjective or objective voice or speech qualities (Table 2) [14, 18, 21]. Subjective and objective voice quality outcomes did not significantly change from pre- to post-surgery times in two studies using endoscopic FTS or TAT [14, 21]. Song et al. [18] reported better postoperative fundamental frequency ranges in patients benefiting from FTS compared with those who benefited from CT.

### Bias analysis

According to MINORS, most authors clearly stated the aim(s) and the endpoints of their respective studies (Additional file 1: Appendix 3). The inclusion of consecutive patients was adequately performed in 5 studies [11, 12, 17, 20, 24], while others conducted retrospective studies and/or did not specify the recruitment features [13–15, 18, 21–23, 26]. In 13 studies [11–14, 16–18, 20–22, 24–26], the endpoint assessment (subjective findings) was performed by surgeons themselves and was not performed independently or in a blinded manner. The outcomes consisted of objective findings in three studies and were, therefore, adequately evaluated according to MINORS [15, 19, 23]. The follow-up period and the loss of follow-up data were not specified in most studies. Because most studies were retrospective or prospective uncontrolled studies, there were no study size calculation, adequate control or contemporary group establishment, or adequate statistical analyses. Precisely, there was no randomized controlled study, leading to low scores of control/contemporary group outcomes in the bias analysis (Additional file 1: Appendix 3).

## Discussion

The present review found that FTS may be performed for benign and malignant lesions, including selected central neck dissection. FTS seems to report comparable outcomes with transaxillary or oral approaches about operative time, complication rates or drainage features, while the conversion rate occurred in 0–6.3% of cases. Many complications were identified, including earlobe hypoesthesia, hematoma, and seroma, but the difficulty to perform adequate controlled randomized trial limits us in the draw of clear conclusion about the advantages and limits of FTS over other surgical approaches.

The number of publications dedicated to robotic thyroid surgery has increased over the past decade as the incidence of small thyroid cancer detection has increased. Some presume that this is due to an increasing number of young women who expect excellent cosmetic results in addition to excellent oncologic outcomes [33]. In this systematic review, we investigated indications, surgical and functional outcomes of endoscopic and robotic FTS. Many points may be highlighted regarding our analysis.

First, there was an important disparity between studies about indications, inclusion and exclusion criteria depending on the experience of teams. Overall, endoscopic and robotic FTS may be performed for hemi [13, 14, 18, 20, 21] and total thyroidectomies [11, 12, 15–17, 19, 22–25], for both benign and limited malignant lesions reaching 6 cm of size [20, 22, 24], with a thyroid lobe volume that does not exceed 10 cm [22]. Depending on studies, the presence of extrathyroidal extension of carcinoma was a criterion of inclusion [15, 16, 18] or exclusion [11, 12, 20–22]. According to several teams [15, 16, 18], FTS may be considered as safe and effective for patients with carcinoma and minimal extrathyroidal extension, which commonly require central neck dissection or dissection of the recurrent laryngeal nerve. For other authors, however [16, 18], the presence of locoregional metastasis results in immediate exclusion from consideration of remote access. As such, the presence of locoregional lymphadenopathy remains an important factor of disparity between studies [11–13, 17, 20–22] although the realization of central node dissection through FTS approach remains feasible. The use of FTS approach for contralateral re-operation seems to be adequate but remains a discussion topic for some teams [16, 18, 19]. Common exclusion criteria included substernal extension, previous neck radiation and advanced thyroid cancer with distant metastases. Future studies aim to establish common FTS recommendations for maximum size of nodules/lobes, BMI threshold, and pathologies (e.g. Graves’ disease, thyroiditis).

Second, the types of surgical and functional outcomes significantly varied between studies. Authors commonly evaluated operative time, hospital stay, drain placement and amount of drainage, as compared with CT [29]. However, much information was lacking in most studies such as console time, docking time, and pocket dissection time [12, 14–16, 18, 20, 22, 23, 25], which limits the comparison with other approaches. Another lacking outcome is the experience of surgeon who performed FTS. The report of the experience of the surgeon(s) is important because there is a consensus that there is a learning curve with remote-access surgery [33, 34]. Many authors did not describe data about blood loss, drain duration or patient comorbidities, which may limit the outcome FTS comparison with CT or other robotic approaches. According to functional and esthetic outcomes, authors agree with the fact that FTS offers the advantage over anterior cervical approaches of completely eliminating neck scar [1, 31]. The esthetic advantage is the most important outcome supporting the development of future thyroid approach but was assessed in few studies [13, 15, 16, 21, 23, 25].

Third, this systematic review data may support the continued finding that remote access surgery has a complication rate comparative with CT. One of the most prevalent postoperative thyroidectomy complications remains transient or permanent laryngeal recurrent nerve injuries, which ranged from 0 to 7.1%, and 0 to 3.4% of FTS cases, respectively. These ranges match with the data of CT [34, 35]. Laryngeal nerve injury may be limited with neuromonitoring [34, 35], which was used by only a few authors in FTS approach [19, 24]. In most studies, laryngeal nerve function was evaluated with postoperative nasopharyngoscopy and subjective assessment of vocal fold movements. The evaluation of vocal fold movement depends on the material used (videolaryngostroboscopy) and the surgeon experience and may, therefore, report low-to-moderate interrater reliability [36, 37]. In practice, it is recommended to assess voice quality through videolaryngostroboscopy, subjective and objective voice evaluations [38], a combination of which was performed in only a few studies [14, 16, 18, 21]. It is commonly accepted that postoperative subtle or perceived voice changes may be related to injury of the superior laryngeal nerve [39], which may reach 50% of patients benefiting from CT [40, 41]. To date, the prevalence of injury of the superior laryngeal nerve was not established in FTS, representing an additional functional outcome that requires future studies. The definition of dysphonia, the use of adequate voice quality evaluation approach, the severity of laryngeal recurrent nerve palsy, and the investigation of laryngeal superior nerve function represent additional outcomes that require future studies.

Seroma and hemorrhage rates varied between studies, and may depend on the placement of drain. However, the comparison of both prevalence and severity of hemorrhages between FTS and CT remains limited because authors did not clearly report the features of hemorrhages, i.e. definition of minor versus major hemorrhages, delay and need of surgery revision. Overall, FTS seems to be as safe as CT approach in terms of postoperative complication rates but future studies need to consider the complication features regarding the surgical approach, surgeon experience, and lesion features (dissection or enlarged thyroidectomy). Moreover, authors have to clearly define the complication and the list of investigated complications to ensure comparison across studies. Indeed, some complications related to robotic approach, e.g. skin flap ischemia, lip weakness, were poorly investigated and, therefore, reported in studies.

The main important limitations of this systematic review are the low number of patients in many studies, the low evidence-based levels of studies, the lack of consideration of many surgical and functional outcomes and the risk of overlap between some studies issue from the same center. The different needs of patients consist of an additional limiting factor. In addition to the disparities between studies, the bias analysis reported that there was no uncontrolled or controlled study with an adequate MINORS score. The lack of randomization process in studies comparing several surgical approaches, the lack of inclusion of consecutive patients, the biased assessment of outcomes and the statistical problems are all issues limiting the draw of clear conclusion. Future studies are needed to determine the limits of FTS in terms of indications, and could compare surgical and functional outcomes between FTS and other approaches.

## Conclusion

FTS is a safe and effective robotic surgical approach for benign and malignant thyroid lesion requiring hemi- or total thyroidectomy with or without central neck dissection. There was an important disparity between studies about inclusion/exclusion criteria, surgical and functional outcomes. FTS appears however to report similar complications to conventional thyroidectomy, excellent cosmetic satisfaction with longer operative times when performed on appropriately selected patients.

## Supplementary Information

**Additional file 1. Appendix 1 footnotes: **The thyroidectomy is performed through the oral vestibule with laparoscopic instruments.

## Data Availability

Data are available on request.

## Abbreviations

CT: Conventional thyroidectomy
FTS: Facelift thyroid surgery
PICOTS: Population, intervention, comparison, outcome, timing and setting
TAT: Transaxillary thyroid surgery
TO: Transoral thyroid surgery
