# Supplementary material for: Facelift thyroid surgery: a systematic review of indications, surgical and functional outcomes
Source: J Otolaryngol Head Neck Surg. 2023 Apr 10;52:25. doi: 10.1186/s40463-023-00624-x (PMC10088190; doi:10.1186/s40463-023-00624-x)
Supplement: Supplementary file 1 — Additional file 1. Appendix 1 footnotes: The thyroidectomy is performed through the oral vestibule with laparoscopic instruments. [file 40463_2023_624_MOESM1_ESM.docx]

**Additional file 1**

**Appendix 1: Example of transoral approach for thyroid surgery.**

**
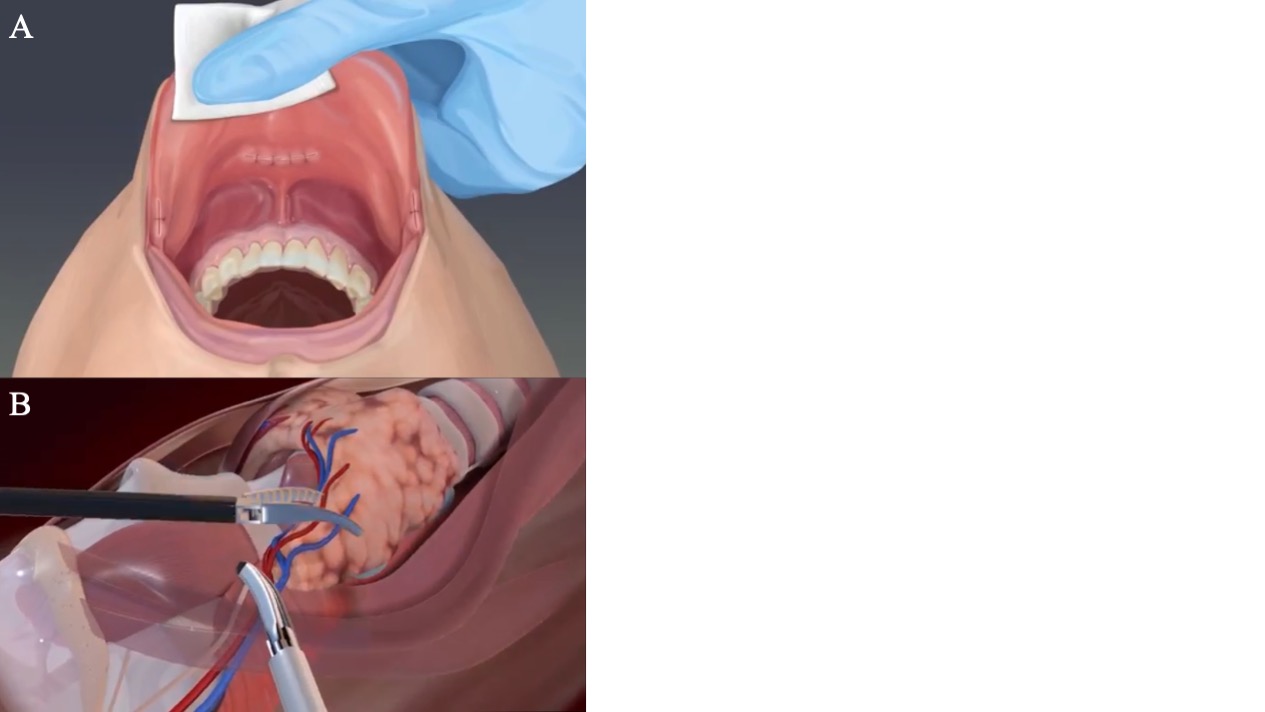
**

**Appendix 1 footnotes:** The thyroidectomy is performed through the oral vestibule with laparoscopic instruments.

**Appendix 2: Characteristics of Studies.**

| **References** | **Design** | **EL** | **Indications & cohort** | **Pathological outcomes** | **Surgical features** | **Complications/Other outcomes** | **Main findings** |
| --- | --- | --- | --- | --- | --- | --- | --- |
| Terris (11) | Prospective | IIIb | Thyroidectomy (hemi/tot) | Benign-Malignant lesion | Operative time: 155 min | Hospital stay: 1 d | 1. PA-R is safe and technically feasible approach |
| 2011, USA | Uncontrolled |  | N=14 PA-R | Tumor size (Gr1-2): 1.7-2cm | Pocket dissection time: 74 min | Seroma: 2 | with minor complications such as seroma or |
|  |  |  | BMI= NP | Papillary cancer: 2 | Docking time: 17.6 min | Transient RNL palsy: 1 | transient earlobe hypoesthesia. |
|  |  |  | F/M=13/1 | Follicular cancer: 1 | Console operative time: 50.5 min | Hypocalcemia: 0 |  |
|  |  |  | Age: 34 yo | Benign lesion: 11 | Drain placement: 1 | Transient earlobe hypoesthesia: 14 |  |
|  |  |  |  | Hemi: 13 | Conversion rate: 0 |  |  |
| Terris (12) | Prospective | IIb | Hemithyroidectomy | Benign-Malignant lesion | Operative time (Gr1-2): | Hospital stay (Gr1-2): 1-1 d | 1. PA-R reported similar operative time, and |
| 2012, USA | Controlled |  | Gr1: 10 PA-R | Tumor size (Gr1-2): 1.7-2cm | 157-196 min | Transient RNL palsy (Gr1-2): 0-0 | better learning curve than TAT approach for |
|  |  |  | Gr2: 15 TAT | Papillary cancer (Gr1-2): 2-1 | Drain placement (Gr1-2): 1-1.1 d | Hematoma (Gr1-2): 0-0 | hemithyroidectomy. |
|  |  |  | BMI (Gr1-2): 26.8-28.5 | Follicular cancer (Gr1-2): 1-0 | Conversion rate: 0 | Transient earlobe hypoesthesia (Gr1): 10 | 2. There was no major complication in both groups. |
|  |  |  | F/M (Gr1-2): 8/2 - 5/0 | Benign (Gr1-2): 7-14 |  |  | 3. Hypoesthesia or earlobe resolved in all patients |
|  |  |  | Age (Gr1-2): 30-45 yo |  |  |  | 6-m post-surgery. |
| Kandil (13) | Prospective | IIIb | Hemithyroidectomy | Benign-Malignant lesion | Operative time: | Hospital stay: 1 d | 1. Retroauricular robotic hemithyroidectomy is |
| 2014, USA | Uncontrolled |  | N=12 PA-R | Tumor size: 1.2 cm | With neck lift surgery: 156 min | Seroma/hypoesthesia: 2-3 | a safe and feasible ambulatory surgery. |
|  |  |  | BMI=28.6 | Benign nodule: 7 | Without neck lift surgery: 145 min | Transient hypocalcemia: 3 | 2. All patients left the hospital on the same day |
|  |  |  | F/M=12/0 | Hashimoto: 2 | Console operative time: 15.2 min | Transient/permanent RNL palsy: 1-0 | of surgery. |
|  |  |  | Age: 45 yo | Papillary cancer: 1 | Docking time: 11.5 min | Permanent hypocalcemia: 0 | 3. All patients reported complete satisfaction |
|  |  |  |  | Follicular cancer: 2 | Blood loss: 22.4 mL | Satisfaction with scar: 12 | after the surgery. |
| Park (14) | Prospective | IIIb | Hemithyroidectomy | Malignant lesion | Operative time: 140 min | Hospital stay: 3.2 d | 1. Retroauricular endoscopic hemithyroidectomy |
| 2014, Korea | Uncontrolled |  | N=11 PA-E +central dissection | Tumor size: 0.5 cm | Drain duration: 3.2 d | Transient earlobe sensory changes: 3 | is a safe approach associated with no major |
|  |  |  | BMI= NP | Papillary cancer: 11 |  | Transient/permanent RNL palsy: 0-0 | complication and similar hospital stay than CT. |
|  |  |  | F/M=11/0 |  |  | GRBAS, F0, Jitt, Shim, NHR, highest F0: | 2. Voice did not change post-surgery (no voice |
|  |  |  | Age: 23-57 yo |  |  | No major post-surgery changes. | outcome statististical analysis). |
|  |  |  |  |  |  |  |  |

| **References** | **Design** | **EL** | **Indications & cohort** | **Pathological outcomes** | **Surgical features** | **Complications/Other outcomes** | **Main findings** |
| --- | --- | --- | --- | --- | --- | --- | --- |
| Byeon (15) | Retrospective | IV | Thyroidectomy (hemi/tot) | Benign-Malignant lesion | Operative time (hemi-tot): 88-174 | Hospital stay (hemi-tot): 6.3-6.5d | 1. PA-R is safe and technically feasible approach |
| 2015, Korea | Chart-review |  | N=87 PA-R | Tumor size: 1.3 cm | Blood loss (hemi-tot): 32-35 | Hematoma/seroma: 2-21 | for hemi- and total thyroidectomy in patients |
|  |  |  | BMI=23.6 | Papillary cancer: 75 | Drainage amount (hemi-tot): | Transient/permanent RNL palsy: 5-3 | with benign or malignant lesions. |
|  |  |  | F/M=67/20 | Follicular cancer: 2 | 142-145 | Mental/spinal nerve lesion: 5-0 | 2. The most common postoperative complications |
|  |  |  | Age: 38 yo | Adenomatous lesion: 9 | Drain duration (hemi-tot): | Transient hypocalcemia: 12 | were seroma, transient hypocalcemia, and |
|  |  |  |  | Follicular adenoma: 1 | 3.3-3.3 d | Earlobe Numbness/chyle leakage: 6-1 | transient RNL. |
|  |  |  |  | Extrathyroidal extension: 40 |  | Skin flap ischemia: 2 |  |
|  |  |  |  | Hemi: 56 |  | Scar satisfaction: 87 |  |
| Sung (16) | Retrospective | IV | Thyroidectomy (hemi/tot) | Benign-Malignant lesion | Operative time: | Hematoma/seroma (Gr1-2): 0-0/3-4 | 1. Both approaches are safe and feasable, reporting |
| 2016, Korea | Chart-review |  | Gr1: 20 PA-R | Tumor size (Gr1-2): 1.2-0.7cm | Total (Gr1-2): 192-169 (Gr1=Gr2) | Permanent RNL palsy (Gr1-2): 0-0 | no significant adverse effects, and similar operative |
|  |  |  | Gr2: 45 TAT | Benign (Gr1-2): 5-7 | Hemi (Gr1-2): 143-135; Gr1=Gr2 | Transient RNL palsy (Gr1-2): 1-0 | outcomes. |
|  |  |  | BMI (Gr1-2): 24.4-23.8 | Malignant (Gr1-2): 15-38 | Amount of drainage: (Gr1=2) | Permanent hypocalcemia (Gr1-2): 0-0 | 2. There were no permanent RNL palsy, hematoma, |
|  |  |  | F/M (Gr1-2): 15/5 - 43/2 | Extrathyroidal extension: 8-18 | Gr1-2: 213-257 mL | Transient hypocalcemia (Gr1-2): 1-2 | hypocalcemia and tracheal injury. |
|  |  |  | Age (Gr1-2): 42 - 50 yo | Stages (Gr1-2) |  | Tracheal injury (Gr1-2): 0-0 | 3. Cosmetic satisfaction and postoperative pain |
|  |  |  |  | I-II-III: 9/30 - 0/0 - 6/8 |  | Cosmesis satisfaction (1- 3w): Gr1=2 | outcomes were comparable across groups. |
|  |  |  |  | Hemi (Gr1-2): 17-29 |  | Postoperative pain (1-, 3d, 1w): Gr1=2 |  |
| Duke (17) | Prospective | IIIb | Thyroidectomy (hemi/tot) | Benign-Malignant lesion | Drain placement: 23 (34) | Outpatient surgery: 63 (62) | 1. FTS is a safe and effective surgical approach |
| 2017, USA | Uncontrolled |  | N=90 (102 PA-R performed) | Benign nodule, cysts, follicular | Pocket dissection time: 68 min | 1-2-3d hospital stay: 35-2-1 | for thyroid surgery. |
|  |  |  | BMI=26.1 | lesions: N=81 (91.1) | Robot positition time: 13.5 min | Incision length: 10.2 cm | 2. Multicenter analysis reported that seroma, |
|  |  |  | F/M=89/1 | Malignant lesion: N=9 (8.9) | Console time: 44.2 min | Postoperative bleeding: 3 | transient RNL, and hematoma are the most common |
|  |  |  | Age: 42 yo | Hemi: N=100 (98) | Total operative time: 162 min | Accessory nerve transient palsy: N=1 | complications. |
|  |  |  |  |  | Conversion rate: 0 | Transient RNL palsy: N=4 |  |
|  |  |  |  |  |  | Hematoma/seroma: N=3-4 |  |
|  |  |  |  |  |  | Hypocalcemia/cellulitis: N=0-1 |  |
| Alshehri (26) | Prospective | IIIb | Thyroidectomy (hemi/tot) | Benign-Malignant lesion | Operative time: 161 min | Hospital stay (0-1-2 nights): 21-17-2 | 1. PA-R and PA-E FTS are safe approaches |
| 2017, USA | Uncontrolled |  | N=37 (32/5 PA-R/E) | Benign nodule: N=21 (57) | Operative time (+neck lift): 189 min | Major hematoma: N=1 | that may be performed in ambulatory in half |
|  |  |  | N=3 parathyroidectomies | Thyroiditis: N=7 (20) | Console time: 16 min | Minor hematoma: N=1 | patients. |
|  |  |  | BMI=26.9 | Malignant lesion: N=9 (23) | Blood loss: 19 mL | Seroma: N=3 | 2. Seroma, hematoma and transient RNL palsy |
|  |  |  | F/M=37/0 | Tumor size: 2.0 cm | Conversion rate: 0 | Transient RNL palsy: N=2 | are the most prevalent complications. |
|  |  |  | Age: 45 yo | Hemi: N=30 (81) |  | Cellulitis: N=1 |  |
|  |  |  |  | Completion: N=7 (19) |  | Hypocalcemia: N=0 |  |

**Appendix 2: Characteristics of Studies.**

| **References** | **Design** | **EL** | **Indications & cohort** | **Pathological outcomes** | **Surgical features** | **Complications/Other outcomes** | **Main findings** |
| --- | --- | --- | --- | --- | --- | --- | --- |
| Song (18) | Prospective | IIb | Hemithyroidectomy | Benign-Malignant lesion | Operative time: Gr1>Gr2 | Hospital stay (Gr1-2): 8.1-8.2d | 1. Some postoperative voice quality outcomes, |
| 2018, Korea | Controlled |  | Gr1: 42 PA-R | Tumor size (Gr1-2): 1.3-1.3 cm | Gr1-2: 146-116 min | Hematoma/seroma (Gr1-2): 0-0/2-4 | such as F0 range, were better in patients who |
|  |  |  | Gr2: 68 CT | Benign (Gr1-2): 12-23 | Amount of drainage: (Gr1=2) | Hypocalcemia (Gr1-2): 1-3 | benefited from PAR hemi-thyroidectomy compared |
|  |  |  | BMI (Gr1-2): 24.9-25.1 | Malignant (Gr1-2): 30-45 | Gr1-2: 122-107 mL | Transient RNL palsy (Gr1-2): 1-2 | with those who had conventional surgery. |
|  |  |  | F/M (Gr1-2): 31/11 - 45/23 | Extrathyroidal extension: 14-20 | Central neck dissection (Gr1-2): | Voice outcomes (1w & 1m): |  |
|  |  |  | Age (Gr1-2): 45 - 55 yo | Benign lesion (Gr1-2): 5-10 | 27-46 | VHI10, I, F0, Jit, Shim, NHR, MPT: Gr1=2 |  |
|  |  |  |  |  |  | Frequency range: Gr1>Gr2 |  |
|  |  |  |  |  |  | Voice outcomes (6m): |  |
|  |  |  |  |  |  | VHI10, I, F0, Jit, Shim, NHR, MPT, F0 |  |
|  |  |  |  |  |  | range: Gr1=2 |  |
| Ban (19) | Retrospective | IV | Thyroidectomy (hemi/tot) | Benign-Malignant lesion | Intraoperative loss of neuro- | Transient RNL palsy (Gr1-2): 4-4 | 1. The neuromonitoring loss and findings were |
| 2018, Korea | Chart-review |  | Gr1: 111 (103 PA-R- 8 PA-E) | Tumor size (Gr1-2): NP | monitoring signal (Gr1-2): 6-4 | Permanent RNL palsy (Gr1-2): 1-1 | comparable between patients who benefited from |
|  |  |  | Gr2: 42 CT | Benign (Gr1-2): 5-3 | Conversion rate: 0 |  | PA-R and those who benefited from CT. |
|  |  |  | BMI (Gr1-2): NP | Malignant (Gr1-2): 106-39 |  |  |  |
|  |  |  | F/M (Gr1-2): 90/21 - 28/14 | Hemi (Gr1-2): 65-11 |  |  |  |
|  |  |  | Age (Gr1-2): 41-54 yo |  |  |  |  |
| Russell (20) | Retrospective | IV | Hemithyroidectomy | Benign-Malignant lesion | Operative time: | Hospital stay (Gr1-2): 1-0.5d | 1. TO and PA-R are safe surgical approaches for |
| 2018, USA | Chart-review |  | Gr1: 20 PA-R | Tumor size (Gr1-2): 3.2-3.6cm | Gr1-2: 201 - 188 min; Gr1=2 | Hypertrophic scaring: | hemi- or total thyroid resection. |
|  |  |  | Gr2: 20 TO | Bethesda I-III (Gr1-2): 17-15 | Drain placement (Gr1-2): 14-2 | Gr1-2: 3-0 | 2. TO resection of thyroid was associared with |
|  |  |  | BMI (Gr1-2): 28.5 - 27.6 | Bethesda IV-VI (Gr1-2): 3-5 | Conversion rate (Gr1-2): 0 - 1 | Transient RNL palsy (Gr1-2): 1-1 | shorter hospital stay than PA-R approach. |
|  |  |  | F/M (Gr1-2): 20/0 - 16/4 |  |  | Permanent RNL palsy (Gr1-2): 0-0 | 3. Surgeons had to place drain more frequently in |
|  |  |  | Age (Gr1-2): 38 - 43 yo |  |  | Hematoma/seroma (Gr1-2): 2-0 | PA-R patients compared with TO patients. |
| Dabas (21) | Prospective | IIIb | Hemithyroidectomy | Benign-Malignant lesion | Pocket dissection time: | Hospital stay: 1.5 d | 1. TAT and PA-R are safe surgical approaches |
| 2018, India | Uncontrolled |  | N=16 (TAT=5 - PA-R=11) | Tumor size: 2.2 cm (<3.5cm) | TAT: 42 min - PA-R: 40 min | VAS pain score: d7>m1>m3 | with low proportion of postoperative compli- |
|  |  |  | BMI=26.1 | Follicular neoplasm: N=9 | Console operative time: | d7: 2; m1: 0.6; m3: 0.25 | cations. Pain and speech score improved from |
|  |  |  | F/M=13/3 | Papillary cancer: N=6 | TAT: 59 min - PA-R: 52 min | Speech score: d7>m1>m3 | 7-day to 3-month postoperative times. |
|  |  |  | Age: 40 yo | Adenomatous lesion: N=1 | Average blood loss: 45 mL | d7: 1.06; m1: 0.5; m3: 0.25 | 3. No patient had postoperative dysphagia, |
|  |  |  |  |  | Conversion rate: 1/16 | Cosmesis satisfaction: 16 | aspiration, shoulder dysfunction or chyle leak. |
|  |  |  |  |  |  | Transient RNL palsy: N=1 | 4. One patient required surgical conversion. |

**Appendix 2: Characteristics of Studies.**

| **References** | **Design** | **EL** | **Indications & cohort** | **Pathological outcomes** | **Surgical features** | **Complications/Other outcomes** | **Main findings** |
| --- | --- | --- | --- | --- | --- | --- | --- |
| Russell (22) | Retrospective | IV | Thyroidectomy (hemi/tot) | Only benign- | Operative time (Gr1-3/4): | Transient RNL palsy | 1. TAT, PA-R and TO are safe surgical approaches |
| 2019, USA | Chart-review |  | Gr1-2: 70 TAT - 54 PA-R | undetermined nodules: | Hemi: 139-179-126-90; Gr1-3>Gr4 | Gr1-4: 1-2-4-6; Gr1-3=Gr4 | for hemi- and total thyroidectomy in patients |
|  |  |  | Gr3-4: 92 TO - 410 CT | Stages: - | Tot: 165-NP-172-126; Gr1-3>Gr4 | Postoperative hh & seroma | with benign or undetermined nodules. |
|  |  |  | Hemi: Gr1-3/4: 169/240 | Tumor size (Gr1-3/4): |  | Gr1-4 (hh): 0-3-0-1; Gr1>2-4 | 2. Operative times are longer in minimal invasive |
|  |  |  | BMI (Gr1-3/4): 27.4-31.7 | 2.8/2.9 cm |  | Gr1-4 (seroma): 2-3-1-2; Gr1=2=3=4 | approaches compared with open surgery. |
|  |  |  | F/M (Gr1-3/4): |  |  |  | 3. Only hematoma proportion was higher in TAT |
|  |  |  | 202/14 / 307/410 |  |  |  | group compared with others. There were no |
|  |  |  | Age (Gr1-3/4): 43.9/53.5 yo |  |  |  | permanent RNL palsy or hypoparathyroidism. |
| Lee (23) | Prospective | IIb | Thyroidectomy (hemi/tot) | Benign-Malignant lesion | TAT: Robotic/endo: 46-4 | Cosmetic satisfaction score | 1. Cosmetic satisfaction and scar consciousness |
| 2020, Korea | Non- |  | Gr1: 50 TAT (27-23-47) | Gr1: 2 (4) - 48 (96) | PA: Robotic/endo: 42-8 | 3- 12-m: Gr1=Gr2>Gr3 | scores were better in patients benefiting from |
|  | Randomized |  | Gr2: 50 PA-R/E (41-9-18) | Gr2: 7 (14) - 43 (86) | Operative time: Gr3>1-2 | Scar consciousness | TAT or PA approaches compared with open |
|  | Controlled |  | Gr3: 50 CT (26-24-38) | Gr3: 10 (40) - 20 (80) | Gr1-2-3: 199-220-159 | 3- 12-m: Gr1=Gr2>Gr3 | cervical thyroidectomy. |
|  |  |  | BMI: 24-25-25 | Stages: I, II, III | Amount of drainage: (Gr1>2>3) | RNL palsy & hypoparathyroidism | 2. Scar healing is worse in TAT and PA groups |
|  |  |  | F/M: 4/46-17/33-11/39 | Tumor size: | Gr1-2-3: 286-170-127 | Gr1-2-3: 0-2-2/2-1-2 | compared with transcervical appraoch group. |
|  |  |  | Age: 40-43.5-57.6 yo | 0.96-1.2-1.08cm |  | Postoperative hh & seroma | 3. There was no permanent RNL palsy. |
|  |  |  |  |  |  | Gr1-2-3: 0-0-0/3-2-1 | 4. There was no conversion to open surgery. |
| Ji (24) | Prospective | IIb | Thyroidectomy (hemi/tot) | Benign-Malignant lesion | NP | Success rate of use of IONM: | 1. It is possible to monitore RLN during PA-R, |
| 2020, Korea | Controlled |  | Gr1: 46 (39PA-R/7PA-E) | Papillary cancer (Gr1-2): 39-46 |  | Gr1-2: 38/46 - 48/58 | PA-E or TO thyroidectomy in the majority of |
|  |  |  | Gr2: 58 TO | Follicular cancer (Gr1-2): 2-1 |  | Transient RNL palsy (Gr1-2): 1-3 | patients. |
|  |  |  | BMI (Gr1-2): NP | Medullary cancer (Gr1-2): 0-1 |  |  | 2. Sensitivy, specificity, positive and negative |
|  |  |  | F/M (Gr1-2): 9/37 - 18/40 | Benign lesion (Gr1-2): 5-10 |  |  | predictive values of IONM were 50, 95, 17, 99%. |
|  |  |  | Age (Gr1-2): 44 - 47 yo | Hemi (Gr1-2): 33-44 |  |  |  |

**Appendix 2: Characteristics of Studies.**

| **References** | **Design** | **EL** | **Indications & cohort** | **Pathological outcomes** | **Surgical features** | **Complications/Other outcomes** | **Main findings** |
| --- | --- | --- | --- | --- | --- | --- | --- |
| Wirth (25) | Prospective | IIb | Thyroidectomy (hemi/tot) | Benign-Malignant lesion | Operative time (Gr1-2-3): | Hospital stay (Gr1-2-3): | 1. PA-E and TAT are both safe and feasible |
| 2021 | Controlled |  | Gr1: 57 PA-E (59 procedures) | Tumor size (Gr1-2-3): NP | 132 - 176 - 152 min; Gr2>Gr1-3 | 2.2-2.7-2.4 d - Gr2 > Gr1-3 | approaches for thyroidectomy. Complication rates |
| Germany |  |  | Gr2: 52 TAT | Benign (Gr1-2-3): 98-50-54 |  | Infections Gr1-2-3: 0-1-0 | are similar at the expcetion of hematoma that |
|  |  |  | Gr3: 111 CT | Malignant (Gr1-2-3): 3-2-5 |  | Temporary numbness (Gr1-2-3): 38-65-63 | wer more prevalent in TAT procedure. |
|  |  |  | BMI: NP | Hemi (Gr1-2-3): 59-10-54 |  | Hematoma (Gr1-2-3): 17-26-43; Gr2>1,3 | 2. Cosmetic results are considered by patients as |
|  |  |  | F/M: 54/5-106/5105/6 |  |  | HH surgical revision (Gr1-2-3): 2-2-4 | excellent (1/6 score) in 84 and 78% of PA-E and |
|  |  |  | Age: 45-48-59 yo |  |  | Transient hypocalcemia (Gr1-3): 1-5-6 | TAT groups; and very good (2/6) in 10 and 18% of |
|  |  |  |  |  |  | Permanent hypocalcemia (Gr1-3): 0-0-0 | same groups, respectively. |
|  |  |  |  |  |  | Transient RNL palsy (Gr1-3): 2-1-3 | 3. Only 8 and 8% of patients of PA-E and TAT |
|  |  |  |  |  |  | Permanent RNL palsy (Gr1-3): 1-1-2 | groups would not choose this techniques again. |
|  |  |  |  |  |  | Cosmetic VAS score (Gr1-2): |  |
|  |  |  |  |  |  | Scores 1-2-3: 43-5-0 / 37-8-1; Gr1=2 |  |

**Appendix 2 footnotes**: Data presented in this table were reviewed by three authors. Abbreviations: BMI=body mass index; CT=conventional cervical approach; F/M=female/male; Endo=endoscopic approach; GRBAS=grade, roughness, breathiness, asthenia and strain; I=intensity; hemi=hemithyroidectomy; HH=hematoma; HS=hospital stay; IONM=intraoperative neural monitoring; MPT=maximum phonation time; ND=neck dissection; NHR=noise-to-harmonic ratio; NP=not provided; PA(-R/E)=post-auricular facelift (robotic/endoscopic); RNL=recurrent laryngeal nerve; TAT=transaxillary thyroidectomy; TO=transoral approach; VAS=visual analog scale; VHI=voice handicap index; yo=years old.

Appendix 3: Bias analysis (MINORS).

|  | Clearly | Inclusion of | Prospective | Endpoints | Unbiased | Follow-up | <5% lost of | Study size | Adequate | Contempo- | Baseline | Adequate | Total |
| --- | --- | --- | --- | --- | --- | --- | --- | --- | --- | --- | --- | --- | --- |
|  | Stated | Consecutive | Data | Appropriate | Endpoint | Adequate | follow-up | Prospective | Control | rary group | equivalence | Statistical | MINORS |
| Authors | Aim | Patients | collection | to study | Assessment | Period |  | Calculation | group |  | of groups | Analyses | Score |
| Terris (11) | 2 | 2 | 2 | 2 | 1 | NP | 2 | 0 | 0 | 0 | 0 | 0 | 11 |
| Terris (12)* | 2 | 2 | 2 | 2 | 1 | 0 | 2 | 0 | 0 | 1 | 2 | 1 | 15 |
| Kandil (13) | 1 | NP | 2 | 2 | 1 | NP | 2 | 0 | 0 | 0 | 0 | 0 | 8 |
| Park (14) | 2 | NP | 0 | 2 | 1 | NP | NP | 0 | 0 | 0 | 0 | 0 | 5 |
| Byeon (15) | 2 | NP | 2 | 2 | 2 | NP | NP | 1 | 0 | 0 | 0 | 0 | 9 |
| Sung (16) | 2 | 0 | 0 | 1 | 1 | NP | NP | 0 | 0 | 1 | 2 | 1 | 8 |
| Duke (17) | 2 | 2 | 2 | 2 | 1 | NP | NP | 0 | 0 | 0 | 0 | 1 | 10 |
| Alshehri (26) | 2 | NP | 2 | 2 | 1 | NP | NP | 0 | 1 | 0 | 0 | 0 | 8 |
| Song (18)* | 2 | NP | 2 | 2 | 1 | 1 | 0 | 0 | 1 | 0 | 1 | 0 | 10 |
| Ban (19) | 2 | 0 | 0 | 2 | 2 | 2 | NP | 0 | 1 | 1 | 0 | 1 | 11 |
| Russel (20) | 2 | 2 | 0 | 2 | 1 | NP | NP | 0 | 1 | 1 | 2 | 1 | 12 |
| Dabas (21) | 2 | 1 | 1 | 2 | 1 | 1 | 2 | 0 | 1 | 0 | 0 | 0 | 11 |
| Russel (22) | 2 | NP | NP | 1 | 1 | NP | NP | 0 | 1 | 1 | 1 | 1 | 8 |
| Lee (23)* | 2 | NP | 2 | 2 | 2 | NP | NP | 0 | 1 | 1 | 1 | 2 | 13 |
| Ji (24)* | 2 | 2 | 2 | 2 | 1 | 1 | NP | 0 | 1 | 0 | 2 | 2 | 15 |
| Wirth (25)* | 2 | 0 | 2 | 1 | 1 | NP | NP | 0 | 1 | 1 | 1 | 2 | 11 |
|  |  |  |  |  |  |  |  |  |  |  |  |  |  |

Appendix 3 footnotes: According to MINORS, the items were scored NP if not provided; 0 if absent; 1 when reported but inadequate; and 2 when reported and adequate. The global ideal score was 16 for non-comparative studies and 24 for comparative* studies [10].
